# Identifying Cleaved and Noncleaved Targets of Small Interfering RNAs and MicroRNAs in Mammalian Cells by SpyCLIP

**DOI:** 10.1016/j.omtn.2020.10.009

**Published:** 2020-10-14

**Authors:** Yao Zhang, Yilan Teng, Wangwen Xiao, Beiying Xu, Ya Zhao, Weihua Li, Ligang Wu

**Affiliations:** 1 NHC Key Lab of Reproduction Regulation (Shanghai Institute of Planned Parenthood Research), School of Pharmacy, Fudan University, Shanghai 200032, China; 2 State Key Laboratory of Molecular Biology, Shanghai Key Laboratory of Molecular Andrology, CAS Center for Excellence in Molecular Cell Science, Institute of Biochemistry and Cell Biology, Chinese Academy of Sciences; University of Chinese Academy of Sciences, Shanghai 200031, China; 3 Jiangsu Key Laboratory of Experimental & Translational Non-coding RNA Research, Institute of Translational Medicine, School of Medicine, Yangzhou University, Yangzhou 225009, China

**Keywords:** RNAi, siRNA, miRNA, off-target, CLIP, SpyTag, AGO2

## Abstract

Recently, the US Food and Drug Administration (FDA) approved the first small interfering RNA (siRNA) drug, marking a significant milestone in the therapeutic use of RNA interference (RNAi) technology. However, off-target gene silencing by siRNA remains one of the major obstacles in siRNA therapy. Although siRNA off-target effects caused by a mechanism known for microRNA (miRNA)-mediated gene repression have been extensively discussed, whether RNAi can cause unintended cleavage through the effector protein AGO2 at sites harboring partially complementary sequences to the siRNA remains unknown. Here, we report a strategy to establish a comprehensive picture of siRNA cleaved and noncleaved off-targets by performing SpyCLIP using wild-type and catalytically inactive AGO2 mutants in parallel. Additionally, we investigated naturally occurring cleavage events mediated by endogenous miRNAs using the same strategy. Our results demonstrated that AGO2 SpyCLIP is a powerful method to identify both the cleaved and noncleaved targets of siRNAs, providing valuable information for improving siRNA design rules.

## Graphical Abstract

**Figure:**
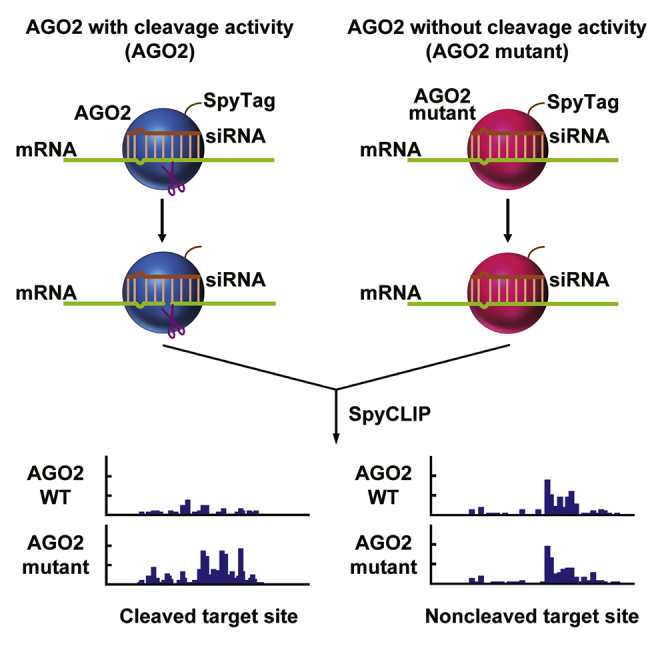

## Teaser

Approval of patisiran and givosiran by the FDA heralds the arrival age of siRNA medicines. However, off-target effect remains a challenge in developing siRNA therapy. Herein, corresponding authors and colleagues report an effective strategy to identify siRNA cleaved and noncleaved off-targets, providing valuable information for improving siRNA design rules.

## Introduction

In mammalian cells, Argonaute (AGO) family proteins associate with small RNAs, including small interfering RNAs (siRNAs) and microRNAs (miRNAs), and assemble with other core components to form the RNA-induced silencing complex (RISC), regulating the expression of target RNAs that contain partially or fully complementary sites to the siRNAs and miRNAs.^1^, ^2^, ^3^, ^4^ Among the four members of the AGO family of proteins expressed in mammals, AGO2 is the only one that exhibits endonuclease activity and cleaves target RNAs that contain fully complementary sites to small RNAs, resulting in RNA interference (RNAi).^5^^,^^6^ In contrast, the ability to mediate translational repression and accelerated deadenylation of the target RNAs harboring partially complementary sequences to the miRNAs and siRNAs is shared by all four mammalian AGOs,^3^^,^^7^ a mechanism known for miRNA-mediated gene repression and siRNA off-target effects.

siRNA has become a powerful tool to silence target genes that has therapeutic potential.^8^^,^^9^ Many RNAi therapeutics using chemically synthesized siRNAs have been undergoing clinical trials for the treatment of various diseases. Patisiran is the first FDA (US Food and Drug Administration)-approved siRNA drug to treat hereditary transthyretin-mediated amyloidosis (hATTR) by silencing transthyretin.^10^^,^^11^ Later, givosiran is approved by the FDA to treat adults with acute hepatic porphyria by silencing aminolevulinate synthase 1 (ALAS1).^12^^,^^13^ More siRNA drugs, such as inclisiran^14^ and ARC-520^15^ to treat hypercholesterolemia and chronic hepatitis B virus infection, respectively, are at different developmental phases. For stable expression in cells, siRNAs can be generated from short hairpin RNA (shRNA) precursors that are transcribed from RNA polymerase III promoters such as U6 and H1.^16^^,^^17^ Alternatively, siRNAs can be produced from a miRNA gene by substituting the mature miRNA sequence with the siRNA sequence via RNA polymerase II promoters.^18^^,^^19^ Despite the difference in their origins, all siRNAs have to incorporate into the four AGO family proteins to execute their functions, thus inevitably causing off-target effects resulting from imperfect base pairing of the siRNAs with the target RNAs; through this mechanism, they can repress nontarget genes via the miRNA-mediated repression pathway,^20^^,^^21^ posing a major challenge for RNAi application in clinical practice. However, whether AGO2-siRNA can also cause unintended cleavage of RNA targets in mammalian cells guided by partially complementary target sites remains unknown.

Although cleaving target mRNAs is known to be the major mechanism by which miRNAs silence gene expression in plants,^22^^,^^23^ only a few cases of cleavage have been reported for endogenous miRNAs in mammals.^24^^,^^25^ Whether AGO2-miRNA-mediated cleavage should be considered an important mechanism for gene regulation in mammalian cells is still being debated. The cleavage product by AGO2 usually undergoes fast decay, which renders the detection of the cleavage intermediates very difficult. To date, global surveys of AGO2-mediated cleavage events in mammalian cells are rare^6^^,^^26^ due to the lack of an accurate experimental approach to determine AGO2-small RNA-target RNA interaction maps.

In this study, we used our recently developed SpyCLIP method,^27^ a covalent link-based crosslinking and immunoprecipitation (CLIP) method with high efficiency and accuracy, along with catalytically inactive AGO2 mutants to systematically examine the siRNA off-targets and the AGO2-mediated target RNA cleavage events within human cells when associated with siRNAs that are produced from shRNA precursors. We also investigated the AGO2-mediated cleavage activities mediated by endogenous miRNAs and revealed the widespread potential miRNA-induced cleavage events in the cells. Our results demonstrated that SpyCLIP using wild-type and cleavage-incompetent AGO2 is a valuable tool for the global study of AGO2-mediated cleavage activities, regardless of whether it associates with exogenous siRNAs or endogenous miRNAs.

## Results

### SpyCLIP Using Wild-Type and Catalytically Inactive AGO2 Proteins Can Identify Cleavage Sites by siRNAs

**Figure 1:**
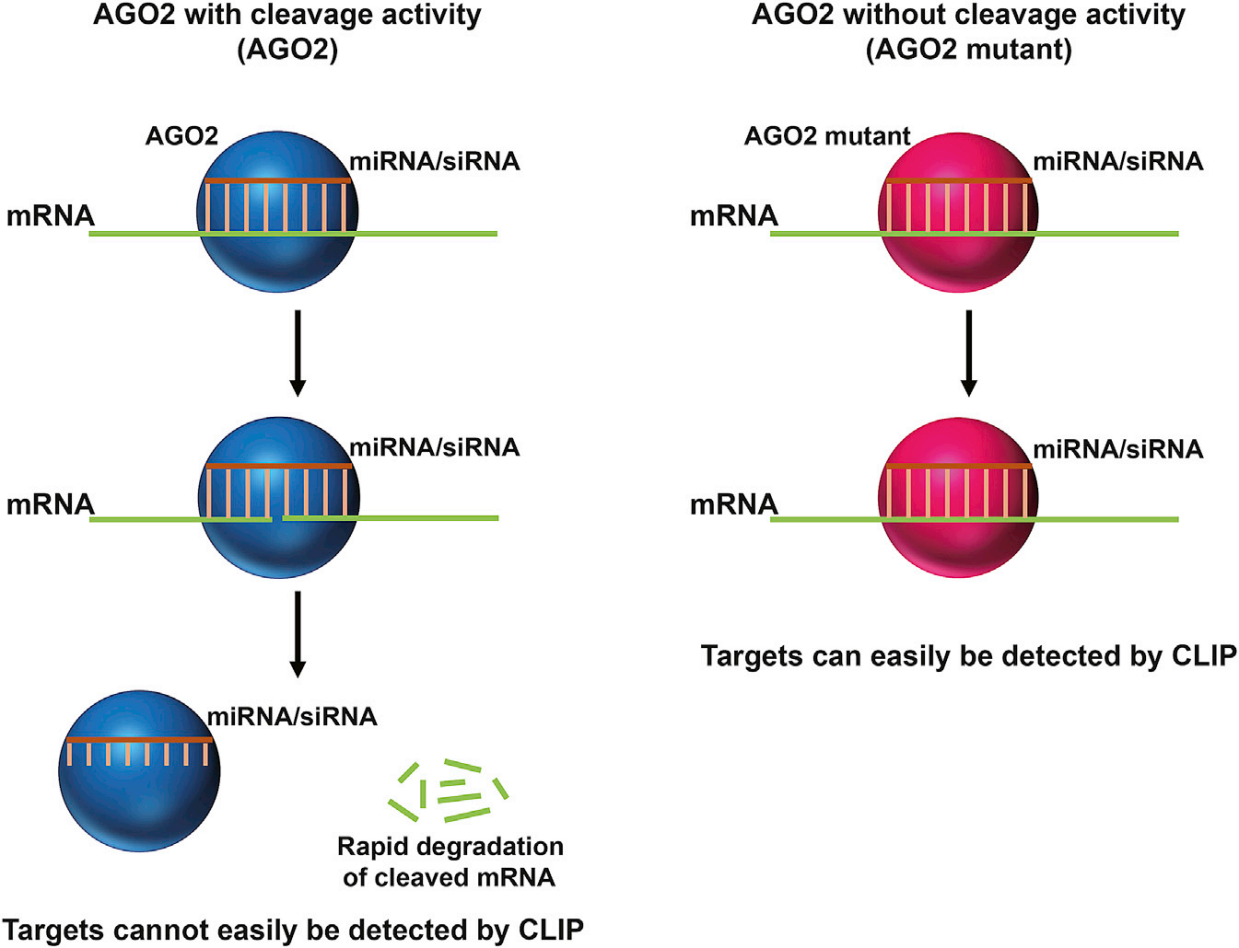
Schematic Representation of the Strategy for Detecting AGO2-siRNA-Mediated Target RNA Cleavage Events by SpyCLIP SpyCLIP against wild-type AGO2 and catalytically inactive AGO2 mutants in the presence of a given siRNA were performed in parallel to facilitate accurate identification of the intermediate RNA fragments cleaved by siRNAs.

In mammalian cells, the AGO2-programmed RISC complex uses siRNA to cleave target RNAs at a discrete position, usually between the 10^th^ and 11^th^ nucleotides downstream of the first nucleotide that was complementary to the siRNA guide strand, and the resultant RNA fragment is then rapidly degraded by exoribonucleases.^28^ This AGO2-siRNA-mediated target RNA cleavage and degradation process is very quick; thus, identification of AGO2-siRNA cleavage sites is a major technical challenge. We hypothesized that a catalytically inactive AGO2-programmed RISC would accomplish siRNA-target RNA recognition and binding without the cleavage event, which would retain the RISC at the binding site and, therefore, permit accurate detection of all binding sites and could distinguish on- and off-target sites compared with those of the wild-type AGO2-programmed RISC counterpart (Figure 1).

**Figure 2:**
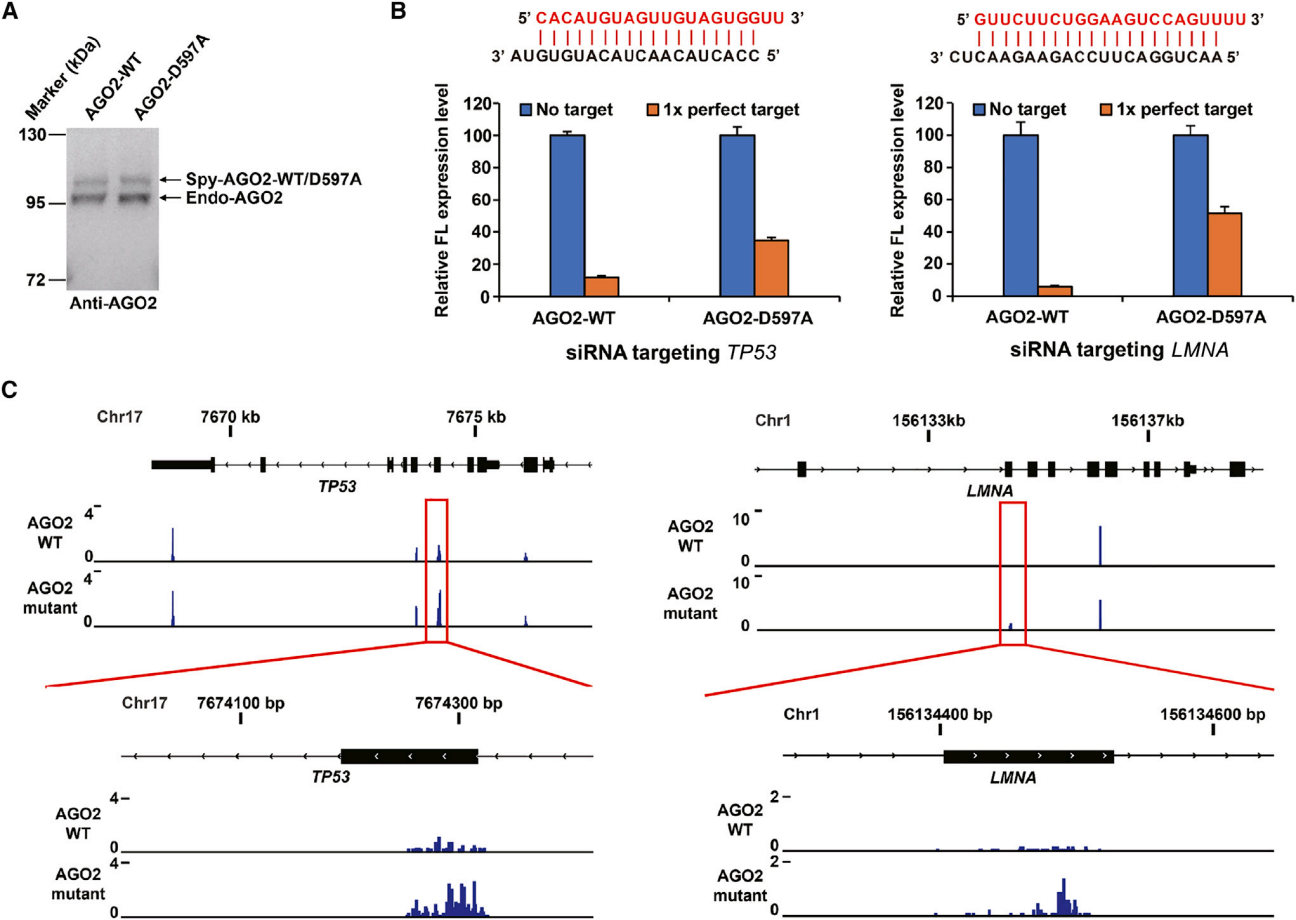
SpyCLIP Generates Highly Accurate AGO2-siRNA On-Target Maps (A) Western blot of endogenous and Dox-induced FLAG-Spy-tagged wild-type or mutant AGO2 proteins. The tagged AGO2 proteins were induced to near-endogenous expression levels by adding 1 ng/mL Dox to the cell culture medium. Both endogenous and induced AGO proteins were probed by anti-AGO2 antibodies. (B) Luciferase reporter assays showing the effectiveness of gene silencing by *TP53* and *LMNA* siRNA produced from the shRNA precursors and the dominant-negative effects of AGO2-D597A. Guide (red) and passenger (black) strand sequences of *TP53* and *LMNA* siRNAs are indicated. Error bars represent the standard deviation of three independent experiments. (C) Read density tracks of SpyCLIP data within the *TP53* and *LMNA* genes. SpyCLIP against wild-type and catalytically inactive AGO2 proteins in the presence of *TP53* or *LMNA* siRNA was performed in parallel to locate the siRNA cleavage site. On-target sites are indicated by red rectangles.

The UV CLIP method is a highly advanced high-throughput tool that can capture all the RNAs that bound to a specific RNA-binding protein with precise location information of the binding sites and has been successfully used in the identification of miRNA-mRNA interaction maps.^29^ Therefore, we adopted our recently developed SpyCLIP to systematically detect the siRNA on- and off-target effects. We first constructed Lenti-X 293T cell lines that stably expressed FLAG- and Spy-tagged AGO2 or its catalytically inactive mutant AGO2-D597A.^5^ The expression of these ectopic AGO2 proteins was induced to near-endogenous levels by adjusting the doxycycline (Dox) concentration (Figure 2A). We then transiently transfected wild-type or mutant AGO2-expressing stable cells with shRNA vectors that produce siRNA sequences targeting the *TP53* or *LMNA* gene (Figure S1). Luciferase reporter mRNAs harboring fully complementary sequences to the siRNAs within the 3′ untranslated region (3′ UTR) demonstrated the strong silencing effects of these siRNAs in wild-type AGO2-expressing cells but the impaired silencing effects in mutant AGO2-expressing cells, which confirmed the effectiveness of these siRNAs and the dominant-negative function of the catalytically inactive AGO2 protein (Figure 2B). Next, we performed SpyCLIP of both the wild-type and catalytically inactive AGO2 proteins in the cells transfected with *TP53*- or *LMNA*-specific shRNA. We found that the siRNA cleavage site on the *TP53* or *LMNA* mRNA can be precisely located by comparing the signal strength of the potential sites identified using wild-type AGO2 and its catalytically inactive counterparts (Figure 2C); efficient cleavage by AGO2-siRNA can dramatically reduce the target mRNA concentration and, therefore, decrease the signal strength of wild-type AGO2 SpyCLIP. The siRNA on-target binding site identified by our strategy exhibited a binding strength that is similar to typical miRNA binding sites in the cells expressing catalytically inactive AGO2 (Figure S2). Thus, we concluded that SpyCLIP using the wild-type and catalytically inactive AGO2 proteins in parallel can faithfully capture the cleavage sites of a given siRNA.

### Detecting Cleaved and Noncleaved siRNA Off-Target Sites of AGO2 by SpyCLIP

**Figure 3:**
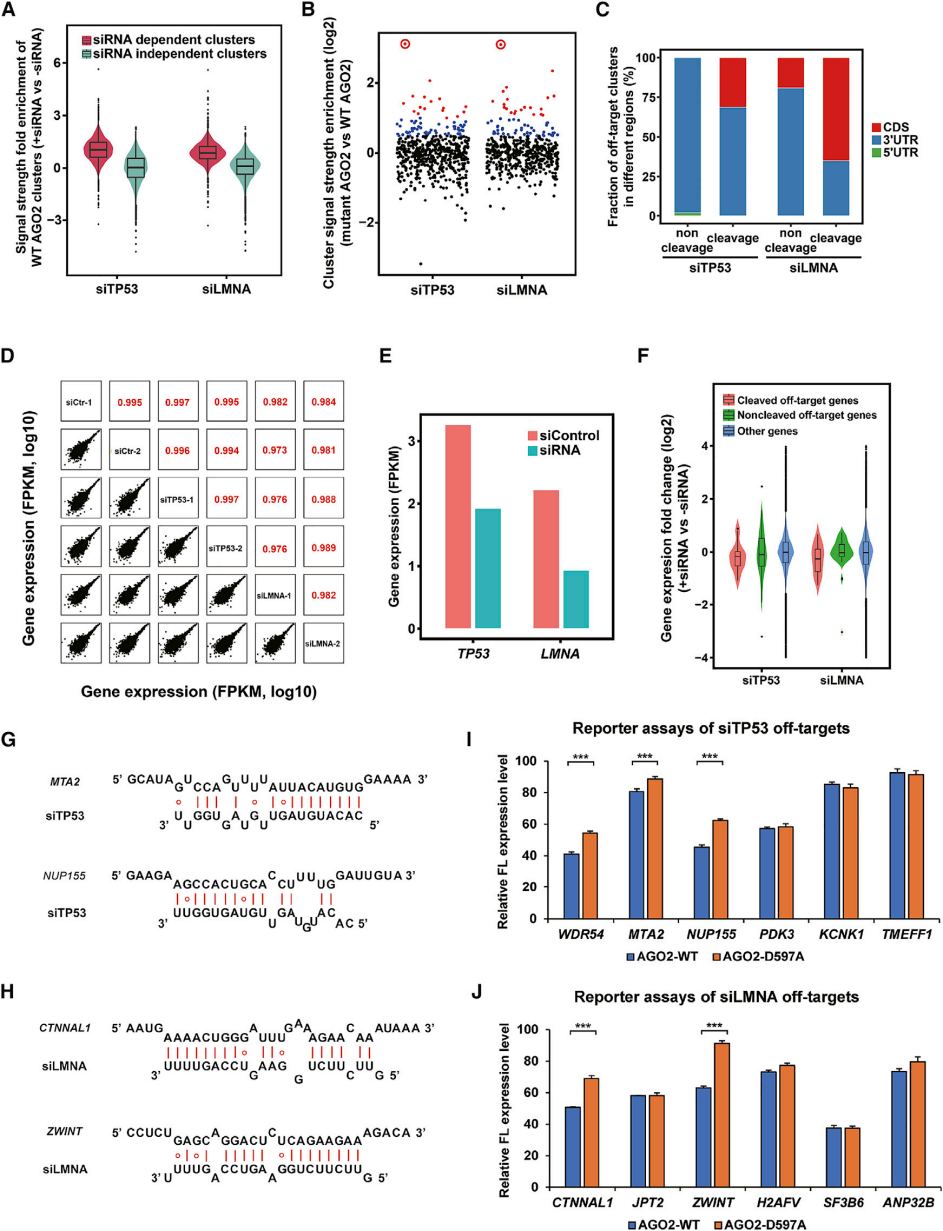
Detection of Cleaved and Noncleaved siRNA Off-Target Sites of AGO2 by SpyCLIP (A) Violin plot representing changes in the read count of the siRNA-dependent SpyCLIP clusters (AGO2-binding sites pool of exogenous introduced siRNAs) and siRNA-independent AGO2 SpyCLIP clusters (AGO2-binding sites pool of endogenous miRNAs) in the cells transfected with shRNA vectors targeting *TP53* (siTP53) or *LMNA* (siLMNA) versus an empty vector (siCtr). (B) Fold enrichment ratio of the read count in the siRNA-enriched clusters from catalytically inactive AGO2 SpyCLIP versus wild-type AGO2 SpyCLIP. The on-target siRNA cleavage sites of *TP53* and *LMNA* are indicated by red circles and are both significantly higher than those of the other clusters. The clusters in catalytically inactive AGO2 SpyCLIP that exhibited 1.4- to 2-fold enrichment over their counterparts in wild-type AGO2 SpyCLIP are indicated by blue dots and defined as noncleaved off-target sites. The clusters in catalytically inactive AGO2 SpyCLIP that exhibited more than 2-fold enrichment over their counterparts in wild-type AGO2 SpyCLIP are indicated by red dots and defined as cleaved off-target sites. (C) Distribution of the *TP53* and *LMNA* siRNA off-target sites in the different regions of protein-coding genes. (D) Abundance comparison of individual genes between cells transfected with shRNA vectors targeting *TP53* (siTP53), *LMNA* (siLMNA), or an empty vector (siCtr). Two biological replicates were performed for each shRNA-expressing cell. (E) RNA sequencing showing that endogenous *TP53* and *LMNA* mRNAs were, indeed, significantly downregulated upon corresponding siRNA treatment. (F) Comparison of fold changes of expression levels of genes with SpyCLIP identified cleaved off-target sites, noncleaved off-target sites, and other genes upon corresponding siRNA transfection. A significant portion of genes bearing cleaved off-target sites were effectively repressed (a major part of the pink violin was below the value 0 of the y axis), whereas only some of the genes bearing noncleaved off-target sites were significantly repressed (green violin). (G and H) Predicted base pairing between the potential cleaved target sites and the guide strand of siTP53 (G) or siLMNA (H). (I and J) Experimental validation of SpyCLIP identified potential cleaved off-target sites of siTP53 (I) or siLMNA (J). Luciferase reporter assays were performed in Lenti-X 293T cells where endogenous AGO2 had been knocked down and replenished with Dox-induced wild-type AGO2 or catalytically inactive AGO2-D597A. Most of the selected off-targets were repressed by corresponding siRNAs in wild-type AGO2-expressing cells, and several of them showed significant de-repression in catalytically inactive AGO2-expressing cells, suggesting that SpyCLIP identified authentic AGO2-cleaved, off-target sites. Error bars represent the standard deviation of three independent experiments. ∗∗∗p < 0.001, Student’s t test.

Measuring the signal strength of each AGO2-siRNA binding site is critical for identifying potential siRNA-mediated off-target sites with high confidence. We first calculated the signal strength of the wild-type AGO2 SpyCLIP clusters that either depend on or do not depend on siRNA expression by comparing the SpyCLIP clusters in the cells transfected with or without shRNAs (Figure 3A). The signal strength of the AGO2-bound sites independent of shRNA expression did not show obvious differences after introducing shRNA, which indicates that introducing siRNA from shRNA-expressing vectors did not cause significant alterations in the AGO2 binding events guided by endogenous miRNAs. To identify the siRNA-related AGO2-bound sites without introducing an artificial bias, we calculated the fold enrichment of the read counts with siRNA expression versus without siRNA expression in each wild-type AGO2 SpyCLIP cluster. Only the clusters exhibiting a fold enrichment higher than the k value^30^ were defined as siRNA-enriched clusters (Figure S3). Following this data-processing pipeline, we identified all siRNA-enriched clusters targeted by the *TP53* and *LMNA* siRNAs and compared the abundance of the siRNA-enriched clusters in the wild-type and mutant AGO2 SpyCLIP. Clusters that were significantly enriched by AGO2 mutant SpyCLIP in the cells transfected with shRNAs were defined as off-target sites.

The on-target sites (indicated by red circles in Figure 3B) for both the *TP53* and *LMNA* siRNAs are the most significantly enriched ones in mutant AGO2 SpyCLIP compared with the wild-type AGO2 counterparts, which was consistent with the model (Figure 1) that RNAs bearing siRNA on-target sites are cleaved by AGO2 and degraded rapidly, whereas RNAs bearing on-target sites bound with catalytically inactive AGO2 remain stable. The majority of the identified siRNA-dependent clusters showed similar signal strength in the wild-type and mutant AGO2 SpyCLIP data (indicated by black dots in Figure 3B). Notably, a portion of the off-target sites had relatively low signal strength in wild-type AGO2 SpyCLIP but significantly higher signal strength in mutant AGO2 SpyCLIP (>2-fold, indicated by red dots in Figure 3B), an interesting feature reminiscent of on-target sites by cleavage mechanism. We proposed that these off-target sites might also undergo AGO2-mediated cleavage events as the on-target site did and defined them as AGO2-cleavage-activity-dependent off-target sites (cleaved off-target sites). The remaining SpyCLIP-identified off-target sites that exhibited 1.4- to 2-fold enrichment over their counterparts in wild-type AGO2 SpyCLIP were defined as AGO2-cleavage-activity-independent off-target sites (noncleaved off-target sites, indicated by blue dots in Figure 3B).

Intriguingly, analysis of the genomic distribution of the *TP53* and *LMNA* siRNA off-target sites showed a significant portion of SpyCLIP-defined cleaved off-target sites located within the coding regions (CDSs) of mRNAs, a region usually occupied by cleavage-competent siRNAs (Figure 3C), which further implies that these cleaved off-targets might be AGO2 cleavage activity dependent. By contrast, the noncleaved off-targets had a strong preference for the 3′ UTRs of protein-coding genes, an action mode similar to that of miRNAs (Figure 3C). Analysis of the base-pairing tendency of these off-target sites with the guide strand of the *TP53* and *LMNA* siRNAs demonstrated a preference for the 5′ half of the siRNA sequence, which corresponded to the seed region of siRNAs (Figure S4). These observations agreed with previous reports showing that the off-target effects of siRNAs are mediated by a miRNA-like mechanism.^31^^,^^32^ In addition, we performed RNA sequencing of cells transfected with different siRNAs and found that endogenous *TP53* and *LMNA* were, indeed, downregulated upon corresponding siRNA treatment (Figures 3D and 3E), suggesting effective siRNA function within the cells. By comparing the fold changes of the expression levels of genes bearing SpyCLIP-identified cleaved and noncleaved off-target sites upon corresponding siRNA transfection, we found that the genes bearing cleaved off-target sites, indeed, exhibited notably higher levels of downregulation than those of the genes bearing noncleaved off-target sites (Figure 3F).

In total, we identified 16 and 19 potential cleaved off-target sites for *TP53* and *LMNA* siRNAs, respectively (Figures S5 and S6). To experimentally verify these potential cleaved off-target sites, six candidates from AGO2-siTP53 or AGO2-siLMNA SpyCLIP-defined cleaved off-target sites were randomly picked for validation. We inserted one copy of the candidate site into the 3′ UTR of a luciferase reporter and compared its expression levels in wild-type AGO2-expressing cells and catalytically inactive AGO2-expressing cells. To prevent endogenous AGO2 from masking the dominant-negative effect of mutant AGO2, we transiently knocked down endogenous AGO2 (Figure S7) by transfecting the cells with a siRNA targeting the 3′ UTR of endogenous AGO2 mRNA, which would not interfere with the expression of AGO2 mRNA bearing a 3′ UTR composed of the herpes simplex virus thymidine kinase (HSV TK) poly(A) signal sequence transcribed from the lentiviral vector. Consistent with our bioinformatic-defined cleaved off-target sites, the majority of these candidates showed marked repression by wild-type AGO2 (indicated by blue bars in Figures 3I and 3J). Three of the siTP53 candidates and two of the siLMNA candidates exhibited significant de-repression upon AGO2 mutant expression (compare blue and orange bars indicated with three asterisks in Figures 3I and 3J), indicating that these sites are responsive to AGO2 cleavage activity. These validated cleaved off-target sites appeared to require more extensive base paring, exhibiting stronger base pairing to the mRNAs in the middle and/or in the 3′ region of the siRNAs (Figures 3G and 3H), consistent with previous studies.^33^ Strikingly, we identified cleaved off-target sites that break the “2–8 seed match” rule for both siRNAs we tested (*NUP155* for siTP53 and *CTNNAL1* for siLMNA; Figures 3G and 3H), which had higher levels of overall base pairing and almost perfect base pairing in the 3′ region, an observation that expands our current understanding of AGO2-small RNA target recognition and repression mechanisms. Also, it should be noted that both the top 1 cleaved off-target candidate for siTP53 and the siLMNA identified by SpyCLIP showed elevated expression levels in RNA-sequencing data (Figures S5 and S6), which provided a typical example that the secondary effects often complicated gene expression alteration-based transcriptome or proteome analysis strategies and highlighted the unique value of developing a straightforward biochemical strategy to capture off-target sites.

### Identifying Endogenous miRNA-Mediated Cleavage Targets in Mammalian Cells

**Figure 4:**
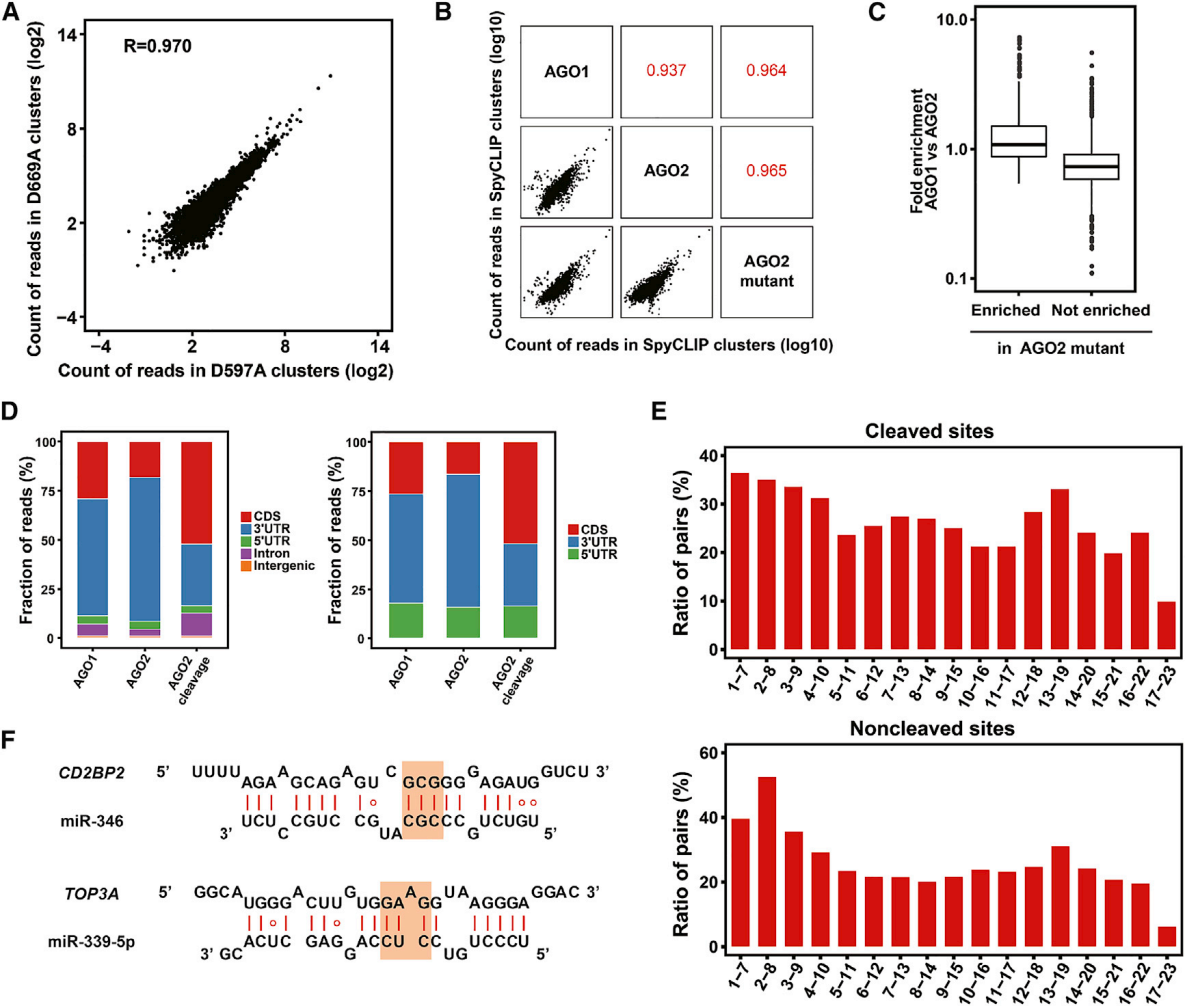
Identification of Endogenous miRNA-Mediated Cleavage Targets in Mammalian Cells (A) The correlation of SpyCLIP reads within the identified clusters of wild-type AGO2 and the AGO2-D597A mutant. (B) The correlation of SpyCLIP reads within the identified clusters of the AGO1, AGO2, and AGO2 mutant samples. (C) Comparison of the fold enrichment in AGO1 SpyCLIP versus AGO2 SpyCLIP between the clusters with or without enrichment in AGO2 mutant SpyCLIP. (D) Distribution of AGO1- and AGO2-bound clusters and endogenous miRNA-mediated cleavage sites in the different regions of the protein-coding genes before and after correction by region size. (E) Base-pairing tendencies of different regions within miRNAs to their complementary sites within SpyCLIP identified miRNA-mediated cleaved or noncleaved target sites. Each 7-mer region (nucleotides 1–7, 2–8, 3–9, and so on) of the AGO2-bound top 100 miRNAs was aligned with AGO2-cleaved or noncleaved target sites identified by SpyCLIP. (F) Two examples of potential miRNA-mRNA cleavage site duplexes. The orange shadows indicate the 9^th^, 10^th^, and 11^th^ nucleotides of the miRNAs whose base pairings with the target sequence were thought to be required for AGO2-mediated cleavage.

To further decipher the AGO2-mediated cleavage events guided by endogenous miRNAs in mammalian cells, we performed SpyCLIP against wild-type AGO2; its two catalytically inactive counterparts, AGO2-D597A and AGO2-D669A; and naturally cleavage-incompetent AGO1 and analyzed the clusters enriched within the catalytically inactive AGOs according to the strategy described in Figure 1A. The clusters identified from AGO2-D597A and AGO2-D669A were almost identical (Figure 4A), which confirmed that SpyCLIP was a highly reproducible tool to generate RNA-binding maps for AGOs. We thus combined the SpyCLIP data of AGO2-D597A and AGO2-D669A, hereinafter referred to as data from the AGO2 mutant, for further analyses.

The majority of clusters identified by SpyCLIP are shared between AGO2 and the AGO2 mutant (defined as AGO2 noncleaved sites), which is consistent with the notion that the miRNA-mediated noncleavage repressive pathway is the dominant function of AGO2 in mammalian cells. Intriguingly, clusters identified by AGO1 SpyCLIP showed a strong correlation with those identified by the AGO2 mutant (Figure 4B). Further analysis demonstrated that the AGO2 mutant-enriched clusters, when compared with those of wild-type AGO2, also exhibited a high enrichment ratio in AGO1 SpyCLIP clusters (Figure 4C). These observations suggest that naturally cleavage-incompetent AGO1 functionally mimics the cleavage-incompetent AGO2 mutant.

Next, we focused on the clusters from AGO1 and AGO2 mutant SpyCLIP that exhibited at least 2-fold enrichment over their counterparts detected in wild-type AGO2 SpyCLIP data and defined them as endogenous AGO2 cleavage sites. The distribution of the clusters enriched in wild-type AGO1 and AGO2 mutant showed a preference for 3′ UTRs, whereas clusters enriched in AGO2-mediated cleaved sites showed a significant preference for the coding regions (Figure 4D), and the distribution pattern did not change when corrected by region size. These results were consistent with the notion that, unlike miRNA-mediated repression that required persistent association of the RISC with the target RNA,^34^ AGO2-mediated cleavage is a process with a fast turnover (cut and run) and, therefore, has an increased ability to target mRNA coding regions despite ribosome trafficking.

We further investigated the base-pairing rules of AGO2-mediated cleavage sites and noncleaved sites. The AGO2-mediated noncleaved sites generally exhibited the highest base-pairing tendency within nucleotides 1–8 of the miRNAs, followed by nucleotides 12–19, consistent with previous results.^27^ However, the AGO2-mediated cleavage sites have less requirement for base pairing within nucleotides 1–8 of the miRNAs but required additional base pairing of the 9^th^, 10^th^, and 11^th^ nucleotides of the miRNAs and extra base pairing in the middle and 3′ regions of the miRNAs (Figure 4E; Figure S8). The duplexes of miR-346 and miR-339-5p and their SpyCLIP-identified potential cleavage target *CD2BP2* and *TOP3A* mRNAs are shown as examples (Figure 4F). The exact base-pairing rule of endogenous AGO2-miRNA-mediated cleavage warrants further investigations. Gene Ontology (GO) analysis of these potential AGO2-miRNA cleaved target RNAs showed enrichment in the processes of DNA topological change, acrosome assembly, and several other critical cellular processes (Figure S9). In summary, AGO2-mediated cleavage of RNAs by endogenous miRNAs might not be as rare as previously estimated. Performing SpyCLIP against wild-type and catalytically inactive AGO2 proteins overcomes the limitation of rapid degradation of cleaved RNA intermediates and provides a convenient option for genome-wide identification of endogenous AGO2 cleavage targets.

## Discussion

The off-target effect of RNAi is one of the major obstacles for its therapeutic applications. miRNA-like off-target effects (cleavage-independent RNA degradation and translational repression) resulting from imperfect base pairing of the siRNAs with nontarget binding sites have been investigated extensively. In addition, unexpected cleavage events are dangerous, because the cleavage mechanism can reduce the abundance of recognized mRNA more significantly by leading to much faster decay of the targets than miRNA-mediated repressive mechanisms. Although the number of AGO2-cleaved off-target sites we identified in this study is lower than that of the miRNA-like noncleaved off-target sites, it should be noted that among the ∼22,000 genes encoded in the human genome, only fewer than 8,000 genes are expressed in each type of cell, and more cleavage-dependent off-target sites would be detected if more cell lines were analyzed. This phenomenon should not be overlooked in future siRNA applications *in vivo*. To date, no reliable experimental method or computational algorithm has been available to effectively identify off-target sites in cells. Although transcriptome or proteome analyses can provide useful information on gene expression alterations in cells transfected with siRNA, genome-wide changes represent a mixture of both primary and secondary effects that are indistinguishable with our current knowledge and, therefore, could not provide sufficient information to improve the design rule for siRNAs. In this study, we reported a straightforward strategy to establish a comprehensive picture of siRNA off-targets by performing SpyCLIP using wild-type AGO2 and catalytically inactive AGO2 mutants in parallel. Using this strategy, we successfully identified many active off-target sites for the siRNA we tested and revealed unexpected base-pairing rules (seed independent) for AGO2-siRNA-guided target cleavage. This strategy is especially valuable for detecting potentially AGO2-cleaved off-targets that are often missed in conventional studies and can be used for routine evaluation of off-targets before siRNA drugs go further into clinical trials.

In recent studies, researchers have made great efforts to introduce various chemical modification on siRNA drugs. For example, the 2′-O-methyl modifications of ribose and the incorporation of phosphorothioate-linked 2′-deoxy-2′-fluoro-modified thymidines at the 3′ ends have been used for patisiran,^10^ and a combination of phosphorothioate, 2′-O-methyl nucleotide and 2′-fluoro nucleotide modifications have been used for inclisiran.^35^ These chemical modifications, indeed, can efficiently suppress siRNA-driven innate immune activation, enhance activity and specificity, and reduce off-target-induced toxicity.^36^ It should be very interesting to use our strategy to investigate whether a chemical modification can effectively reduce cleaved and noncleaved off-target sites.

Endogenous miRNA-meditated cleavage events have long been a subject of debate in mammalian cells. Our study has identified many potential endogenous cleavage events related to several important cellular processes. Unlike the classical miRNA target sites that are mainly located in the 3′ UTR, these potential cleavage sites could be located in both the CDSs and UTRs and tend to require extensive base pairing with their target RNAs, which is consistent with the known mechanisms of siRNA-mediated cleavage.

In summary, a parallel study of wild-type and catalytically inactive AGO2 binding maps using SpyCLIP provides an efficient strategy to investigate AGO2-dependent cleavage events of siRNAs or miRNAs genome-wide in cells as well as to select siRNAs with the fewest off-target effects and help establish improved rules for siRNA design.

## Materials and Methods

### Plasmid Construction

The Dox-inducible lentiviral vector containing N-terminal sequences encoding a 3× FLAG tag (DYKDHDGDYKDHDIDYKDDDDK), a PreScission Protease site (LEVLFQGP), and a SpyTag (AHIVMVDAYKPTK) was described previously.^27^ The CDSs of AGO1, AGO2, AGO2-D597A, and AGO2-D669A were subcloned into the aforementioned lentiviral vector via AgeI and PacI restriction sites from in-house constructed plasmids. Plasmids for the expression of shRNA were the same as those described in a previous publication.^37^ The siTP53 and siLMNA on- and off-target validation plasmids were constructed by inserting one copy of the identified target site into an in-house-made dual luciferase reporter vector that expresses both a firefly luciferase transcript harboring one target site in its 3′ UTR and a control Renilla luciferase transcript.^34^

### Cell Culture and Stable Cell Lines

Lenti-X 293T (Clontech, Mountain View, CA, USA) cells were grown in Dulbecco’s modified Eagle’s medium (Clontech, Mountain View, CA, USA) with 10% fetal bovine serum (GIBCO, Chagrin Falls, OH, USA). For production of the lentiviruses, Lenti-X 293T cells were transfected with a virus vector encoding an AGO protein as well as the VSVG and ΔR8.91 plasmids by using Lipofectamine 2000 (Invitrogen, Carlsbad, CA, USA). Lentiviruses were harvested 48 h post-transfection. AGO-expressing Lenti-X 293T stable cell lines were generated by transduction with lentiviruses in the presence of 8 μg/mL polybrene (Sigma-Aldrich, St. Louis, MO, USA) overnight, followed by selection with 1 μg/mL puromycin (BBI, Shanghai, China) for 1 week. For AGO2 SpyCLIP experiments, wild-type and mutant AGO2 proteins were induced to near-endogenous expression levels by adding 1 ng/mL Dox (BBI, Shanghai, China). For validation of potential AGO2-cleaved targets using a dominant-negative strategy, wild-type and mutant AGO2 were induced by adding 10 ng/mL Dox to produce an excessive amount of proteins.

### Luciferase Reporter Assays

For validation of siTP53 and siLMNA on-target sites, Lenti-X 293T cells that stably expressed 3FPS-AGO2 or 3FPS-AGO2-D597A were plated into 24-well plates and transiently transfected with 500 ng of the shRNA-expressing plasmids, together with 50 ng of a dual luciferase reporter harboring one perfectly complementary target site to the siRNA or a control scrambled site in the 3′ UTR. For validation of siTP53 and siLMNA off-target sites, Lenti-X 293T cells that stably expressed 3FPS-AGO2 or 3FPS-AGO2-D597A were plated into 60-mm dishes and transiently transfected with 200 pmol of siRNAs targeting the 3′ UTR of endogenous AGO2 mRNA. After 24 h, the cells were re-plated into 24-well plates and transiently transfected with 300 ng of the shRNA-expressing plasmids, together with 100 ng of a dual luciferase reporter harboring one copy of SpyCLIP-identified off-target site to the corresponding siRNA or a control scrambled site in the 3′ UTR. The cells were harvested in 100 μL passive lysis buffer 48 h post-transfection. The firefly and Renilla luciferase activities were measured using the Dual-Luciferase Reporter Assay System (Promega, Madison, WI, USA).

### Quantitative Real-Time PCR Assays

To test the knockdown efficiency of endogenous AGO2, Lenti-X 293T cells were plated into 60-mm dishes and transiently transfected with 200 pmol siRNAs targeting the 3′ UTR of endogenous AGO2 mRNA or 200 pmol negative control siRNAs (GenePharma, Shanghai, China). Total RNA was extracted 48 h post-transfection. 300 ng RNA was reverse transcribed using M-MLV reverse transcriptase (RT) (Takara, Otsu, Shiga, Japan), according to the manufacturer’s instructions. The RT products were diluted 10-fold and subjected to quantitative real-time PCR. Real-time PCR was performed on a StepOnePlus Real-Time PCR System (Applied Biosystems, Foster City, CA, USA) with in-house-made SYBR Green PCR Master Mix. The PCR mixtures were heated to 95°C for 1 min and then subjected to 40 amplification cycles (10 s at 95°C, 15 s at 55°C, 30 s at 72°C). The siRNA sequences targeting endogenous *TP53* (siTP53-3′ UTR), scrambled negative control siRNA (siNC) sequences, and the qPCR primer sequences were as follows:

siTP53-3′ UTR-sense: 5′-UAGAAUCUCAAAGCUGCGCAG-3′;
siTP53-3′ UTR-antisense: 5′-GCGCAGCUUUGAGAUUCUAGG-3′;
siNC-sense: 5′-UUCUCCGAACGUGUCACGUTT-3′;
siNC-antisense: 5′-ACGUGACACGUUCGGAGAATT-3′;
AGO2-qPCR+: 5′-TTCAAGGACAGGCACAAG-3′;
AGO2-qPCR−: 5′-CCTGCCACAATGTTACAGA-3′;
GAPDH-qPCR+: 5′-AGTCCACTGGCGTCTTCACC-3′; and
GAPDH-qPCR−: 5′-TGAGGCTGTTGTCATACTTC-3′.

### SpyCLIP of AGO Proteins

AGO-siRNA complex purification and library construction were conducted according to our previous publication.^27^

### High-Throughput Sequencing and Mapping

High-throughput sequencing of the SpyCLIP libraries was performed on the Illumina HiSeq X10 platform and processed as previously described.^26^ All the mapped reads were visualized on the Integrative Genomics Viewer (IGV). All the sequencing data have been deposited in the National Center for Biotechnology Information Gene Expression Omnibus under accession number GSE145438.All the sequencing data have been deposited in the National Center for Biotechnology Information Gene Expression Omnibus under accession number GSE145438.

### Identification of SpyCLIP Clusters and Noise Deduction

Because the 5′ end of the insert cDNA was the crosslinking site of the RNA-binding protein, the clusters were identified based on the 5′ start position of usable reads by using the iCount program (https://github.com/tomazc/iCount)^33^ with 6-nt clustering. The number of usable reads in each cluster was normalized to RPM (reads per million genome mapped reads) and considered the abundance. The correlation of different SpyCLIP libraries was determined by comparing the abundances of all identified clusters in these samples. The SpyCLIP clusters were normalized against the clusters identified in the input library to remove noise signals. The relative abundance of each cluster in SpyCLIP and the input sample was calculated. The SpyCLIP clusters exhibiting more than 5 RPM and at least 15-fold enrichment over their corresponding input counterparts were defined as RNA-binding-protein-specific clusters.

### Identification of siRNA-Enriched Clusters and siRNA Target Sites

The identified AGO2-specific clusters with or without shRNA expression were combined; the abundance of each cluster with or without shRNA expression was counted, and the fold enrichment ratio of each cluster was also calculated. Only those clusters exhibiting fold enrichment over the k value whose density was considerably lower than that of the clusters with lower fold enrichment ratio were defined as siRNA-enriched clusters. The abundance of siRNA-enriched clusters in wild-type and mutant AGO2 SpyCLIP was then calculated. The cleaved and noncleaved off-target sites were identified by the relative signal strength enrichment of each siRNA-enriched cluster in the wild-type and mutant AGO2 SpyCLIP data. Clusters that exhibited at least 2-fold enrichment over the median in siRNA-enriched clusters were defined as siRNA cleaved off-target sites. Clusters exhibited 1.4- to 2-fold enrichment were defined as siRNA noncleaved off-target sites.

### Identification of Endogenous miRNA-Mediated Cleavage Target Sites

The identified mutant AGO2 (D597A and D660A) and wild-type AGO2 SpyCLIP clusters were combined. The abundance of each cluster in AGO1 SpyCLIP, mutant AGO2 SpyCLIP, and wild-type AGO2 SpyCLIP was calculated. Clusters in AGO1 and mutant AGO2 SpyCLIP that exhibited at least a 2-fold increase over their counterparts in wild-type AGO2 SpyCLIP were defined as endogenous miRNA-mediated cleavage target sites.

### Prediction of siRNA/miRNA Target Sites within the AGO2 SpyCLIP Clusters

The corresponding siRNA or the top 100 miRNAs identified by AGO2 immunoprecipitation (IP)^27^ were selected, and successive 7-mer sequences along each siRNA or miRNA were used to search for complementary targets in AGO2 SpyCLIP clusters. These 7-mer sequences were classified by the starting position in the siRNA or miRNA. Base pairing of siRNA or miRNA with its target site was predicted by the RNAhybrid program (v.2.1.2).

### Detection of Gene Expression Fold Change upon siRNA Treatment by RNA Sequencing

The adaptor segment at the 3′ end of the sequenced reads was removed using the FASTX program (v.0.0.14) (-l 1 -c). The remaining reads were mapped to the human genome (v.hg38) using the HISAT program (v.0.1.6) (-no-unal–rna-strandness FR–fr), and the gene expression level was determined by the StringTie program (v.1.3.5). The gene expression fold change upon siRNA treatment was calculated by comparing the gene expression levels in samples transfected with a shRNA targeting *TP53*, *LMNA*, or an empty vector.

## Author Contributions

Y. Zhao, W.L., and L.W. conceived and designed the study. Y. Zhang performed computational analyses. Y.T., B.X., and W.X. performed experiments. Y. Zhao, W.L., and L.W. wrote the manuscript.

## Conflicts of Interest

The authors declare no competing interests.

## Supplemental Information

Document S1. Figures S1–S9

Document S2. Article plus Supplemental Information
