# Supplementary material for: Identifying Cleaved and Noncleaved Targets of Small Interfering RNAs and MicroRNAs in Mammalian Cells by SpyCLIP
Source: Mol Ther Nucleic Acids. 2020 Oct 14;22:900–9. doi: 10.1016/j.omtn.2020.10.009 (PMC7666362; doi:10.1016/j.omtn.2020.10.009)
Supplement: Document S1. Figures S1–S9 [file mmc1.pdf]

OMTN, Volume *22*

Supplemental Information

Identifying Cleaved and Noncleaved Targets of Small Interfering RNAs and MicroRNAs in Mammalian Cells by SpyCLIP

Yao Zhang, Yilan Teng, Wangwen Xiao, Beiying Xu, Ya Zhao, Weihua Li, and Ligang Wu

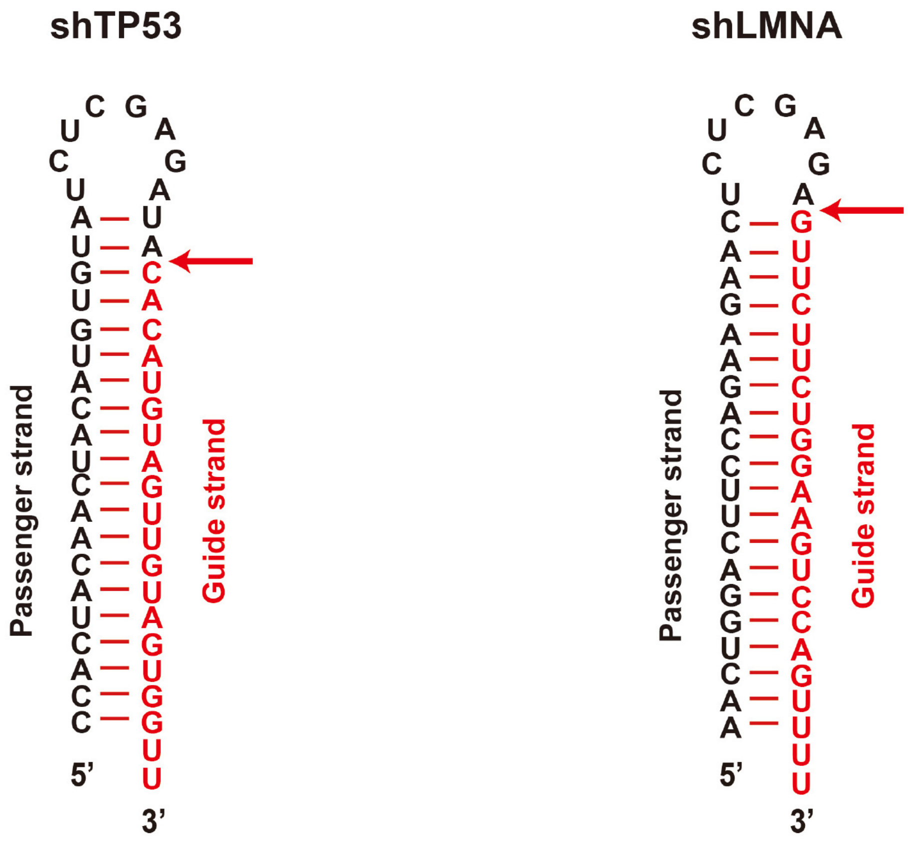

Figure S1. Sequences of *TP53* and *LMNA* shRNAs

Processing of shTP53 and shLMNA was analyzed by deep sequencing. The 5’ end of the most abundant isoform of the siRNA guide strand is indicated by a red arrow.

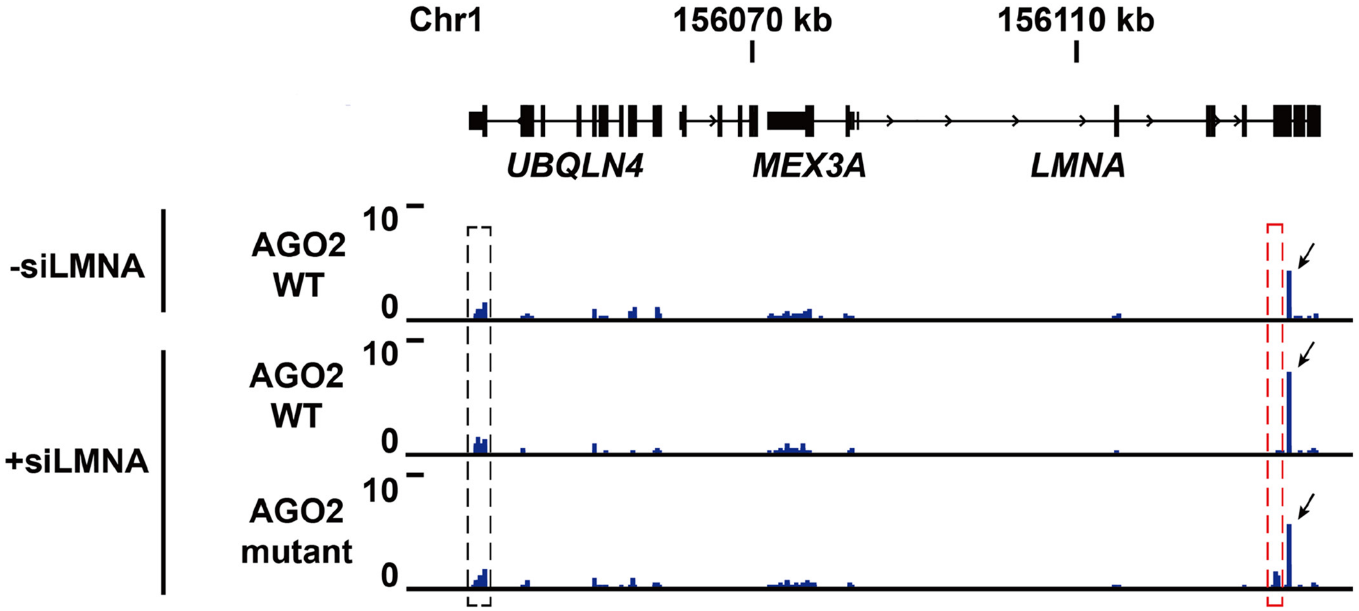

Figure S2. The on-target site of siLMNA exhibits comparable read count with a typical endogenous AGO2-miRNA binding site in cells expressing catalytically inactive AGO2

Read density tracks of AGO2 SpyCLIP data with or without siLMNA treatment. The siLMNA on-target site is indicated by the red dashed rectangle, and a region containing the let-7 miRNA family binding site is indicated by the grey dashed rectangle. The cluster (indicated by an arrow) downstream of the siLMNA on-target site was located in the intron region, which is not a rational binding region for miRNAs in the cytoplasm. In addition, it also existed in AGO2 SpyCLIP without siRNA expression (-siLMNA, AGO2 WT), suggesting that this cluster is irrelevant to the introduced siRNA and is more likely to be a background noise.

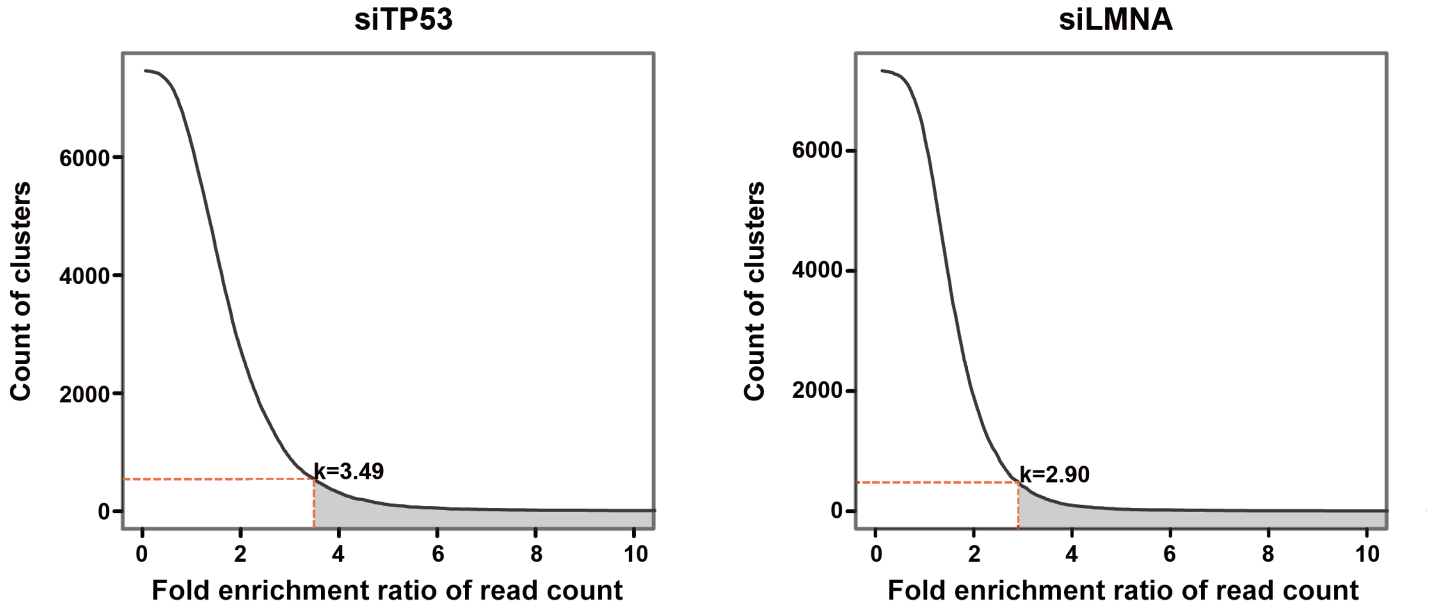

Figure S3. Identification and definition of siRNA-enriched clusters

A cumulative distribution plot showing the fold enrichment ratio of the read count in the wild-type AGO2 SpyCLIP clusters with siRNA expression versus without siRNA expression. The X-axis plots the fold enrichment ratio of the read count in the AGO2 SpyCLIP clusters, and the Y-axis plots the corresponding count of the clusters that exhibit a fold enrichment ratio of the read count above the X-axis value. The fold enrichment ratio at the inflection point of the curve is defined as the k-value. The distribution density of the clusters whose fold enrichment ratio is larger than k is considerably lower than that of the clusters whose fold enrichment ratio is less than k, and these clusters that exhibit an enrichment ratio over the k-value are defined as the siRNA-enriched clusters (grey area).

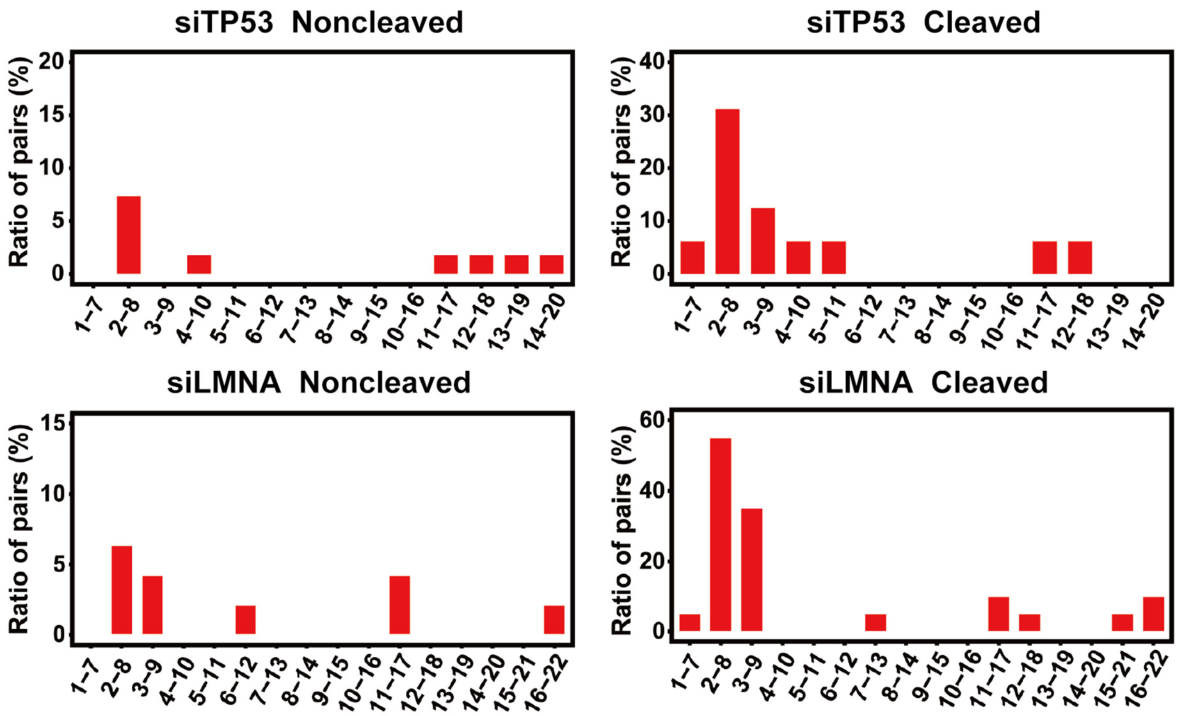

Figure S4. The siRNA-mediated cleaved and noncleaved sites exhibit base-paring preference for the seed region

Base-paring tendencies of the regions within the siRNA guide strand to their complementary sites within the identified cleaved and noncleaved off-target sites. Each 7-mer region (nucleotides 1-7, 2-8, 3-9, and so on) of the *TP53* or *LMNA* siRNA was aligned ± 20 nt around the AGO2-bound off-target sites identified by SpyCLIP.

Figure S5

	AGO2 SpyCLIP cluster strength enrichment ratio (D597A vs WT)	Gene expression fold change (siTP53 vs siControl)
**WDR54** 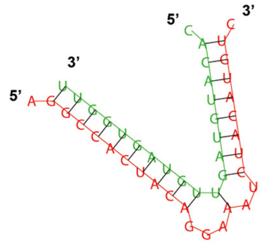	**4.178**	**1.834**
**CUL2** 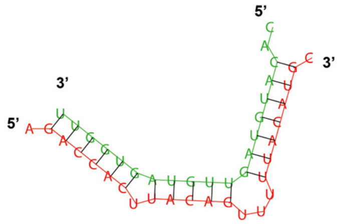	**3.076**	**0.937**
**MTA2** 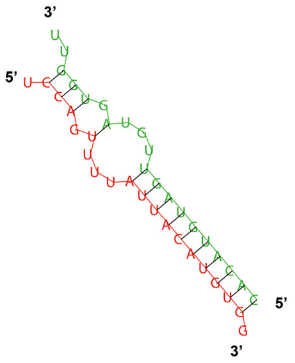	**3.026**	**0.780**
**NUP155** 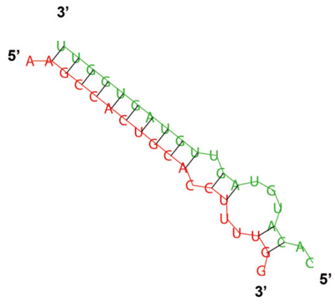	**2.923**	**0.693**
**IDS** 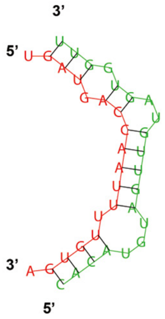	**2.766**	**0.707**

	AGO2 SpyCLIP cluster strength enrichment ratio (D597A vs WT)	Gene expression fold change (siTP53 vs siControl)
**SLC39A10** 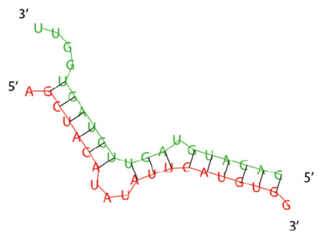	2.669	0.855
**MOSPD2** 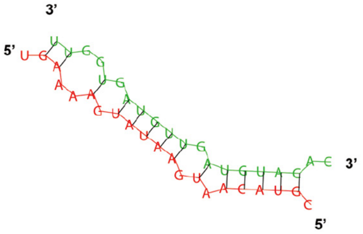	2.499	1.023
**TOMM70** 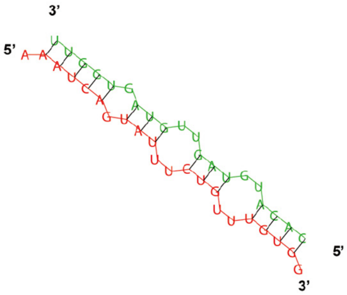	2.467	0.964
**PDK3** 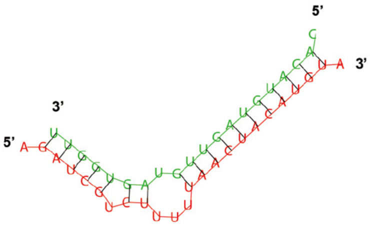	2.432	0.470
**KCNK1** 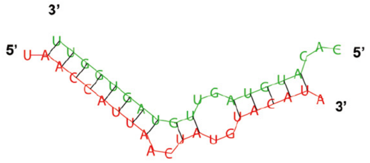	2.406	0.676
**NXPE3** 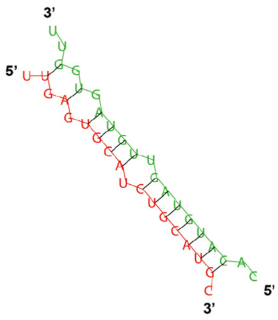	2.325	1.734

	AGO2 SpyCLIP cluster strength enrichment ratio (D597A vs WT)	Gene expression fold change (siTP53 vs siControl)
**TMEFF1** 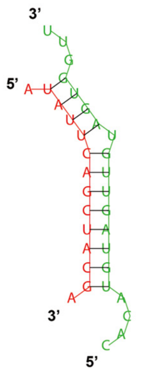	2.282	0.657
**RO60** 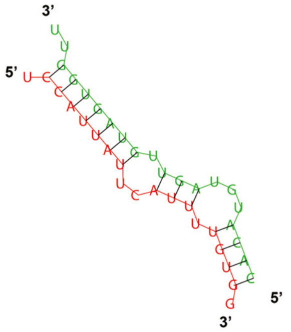	2.252	1.284
**PGM2L1** 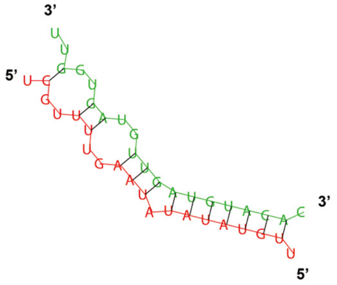	2.173	0.514
**THRAP3** 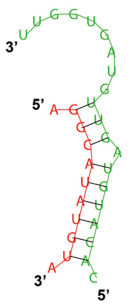	2.056	1.000
**HSF2** 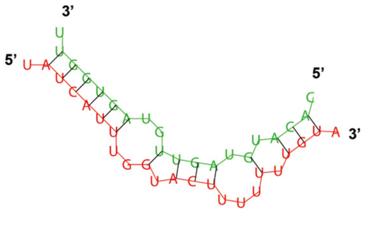	2.004	0.913

Figure S5. A list of SpyCLIP identified siTP53 cleaved targets

Duplexes predicted for the potential AGO2 cleaved target sites base-paired with the guide strand of siTP53 are shown in the left column. AGO2 SpyCLIP cluster strength enrichment ratios (mutant versus wild-type) are shown in the central column. Expression fold changes (siTP53 versus siControl) of these potential cleaved targets are shown in the right column.

Figure S6

	AGO2 SpyCLIP cluster strength enrichment ratio (D597A vs WT)	Gene expression fold change (siLMNA vs siControl)
**CTNNAL1** 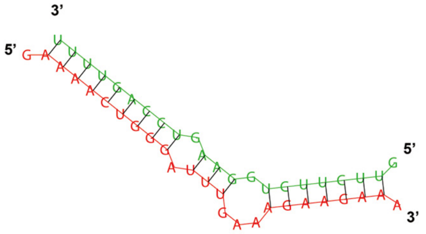	**5.075**	**1.576**
**ACBD3** 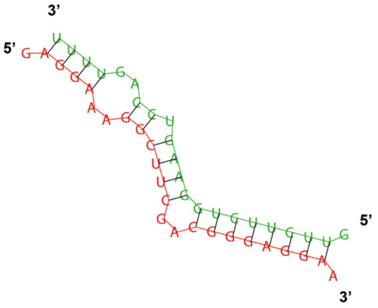	**3.670**	**0.778**
**LSM6** 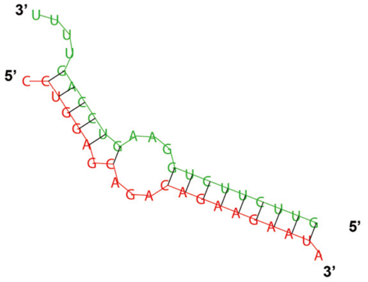	**3.388**	**1.035**
**JPT2** 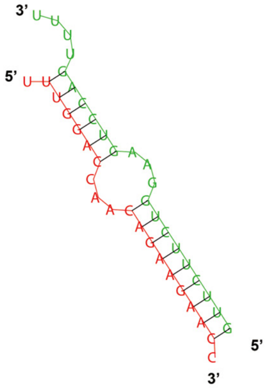	**3.082**	**0.419**
**ZWINT** 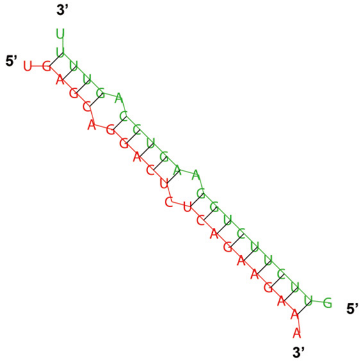	**2.981**	**0.718**

	AGO2 SpyCLIP cluster strength enrichment ratio (D597A vs WT)	Gene expression fold change (siLMNA vs siControl)
**TOP2A** 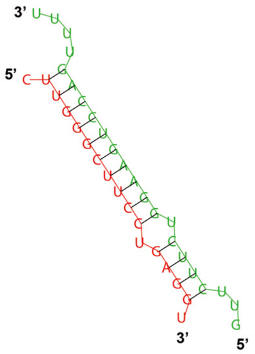	**2.893**	**0.821**
**JPT2** 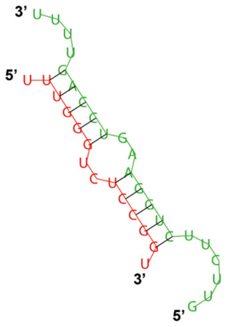	**2.775**	**0.419**
**RADX** 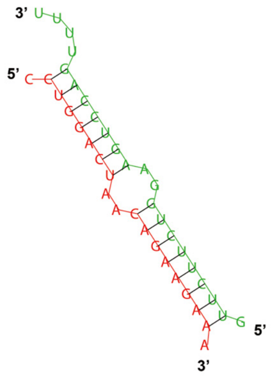	**2.611**	**1.132**
**H2AFV** 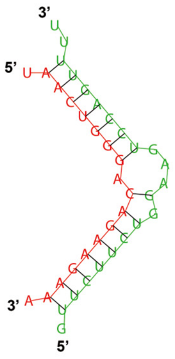	**2.468**	**0.510**

	AGO2 SpyCLIP cluster strength enrichment ratio (D597A vs WT)	Gene expression fold change (siLMNA vs siControl)
**ZC3H13** 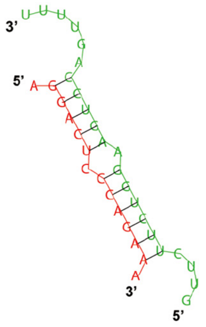	**2.460**	**0.538**
**SF3B6** 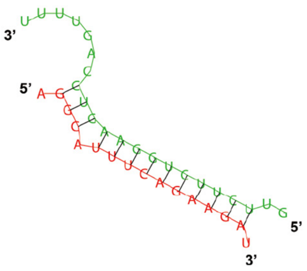	**2.402**	**0.565**
**NUSAP1** 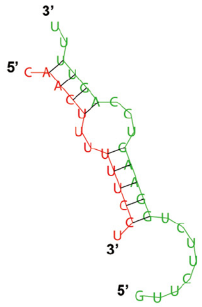	**2.389**	**1.078**
**NUDT3** 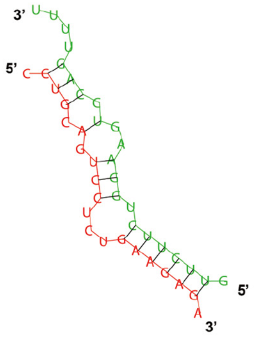	**2.347**	**1.001**
**HMGCR** 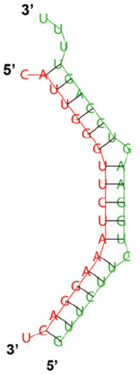	**2.284**	**1.334**

	AGO2 SpyCLIP cluster strength enrichment ratio (D597A vs WT)	Gene expression fold change (siLMNA vs siControl)
**TLK1** 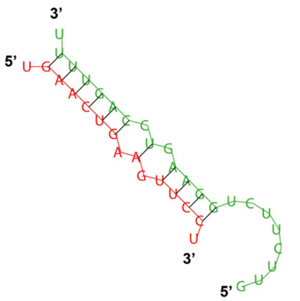	**2.227**	**0.995**
**TP53BP2** 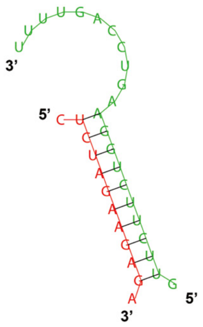	**2.183**	**0.682**
**NBN** 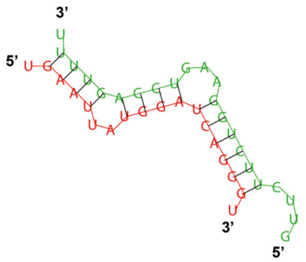	**2.147**	**1.369**
**AAMP** 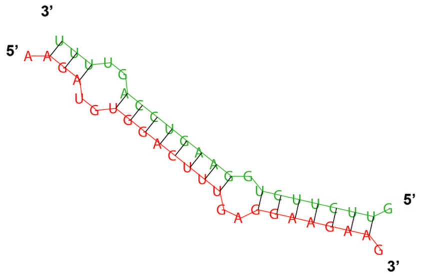	**2.146**	**0.832**
**ANP32B** 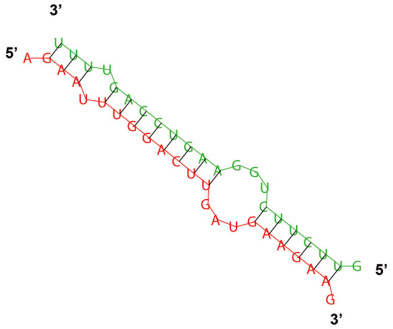	**2.114**	**0.498**

Figure S6. A list of SpyCLIP identified siLMNA cleaved targets

Duplexes predicted for the potential AGO2 cleaved target sites base-paired with the guide strand of siLMNA are shown in the left column. AGO2 SpyCLIP cluster strength enrichment ratios (mutant versus wild-type) are shown in the central column. Expression fold changes (siLMNA versus siControl) of these potential cleaved targets are shown in the right column.

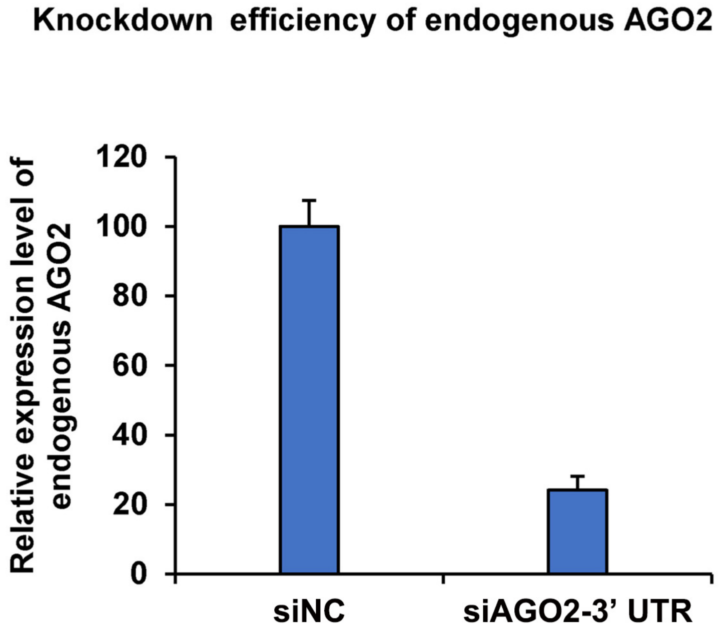

Figure S7. Validation of efficient knock down of endogenous AGO2 by qRT-PCR

Lenti-X 293T cells were transfected with a siRNA targeting the 3' UTR of endogenous *AGO2* (siAGO2-3' UTR) or a scrambled control siRNA (siNC). Total RNA was extracted from the cells 48 hr post transfection, and the abundance of *AGO2* and *GAPDH* mRNA were determined by qRT-PCR. The relative *AGO2* mRNA level was calculated by normalizing to *GAPDH* mRNA.

Figure S8

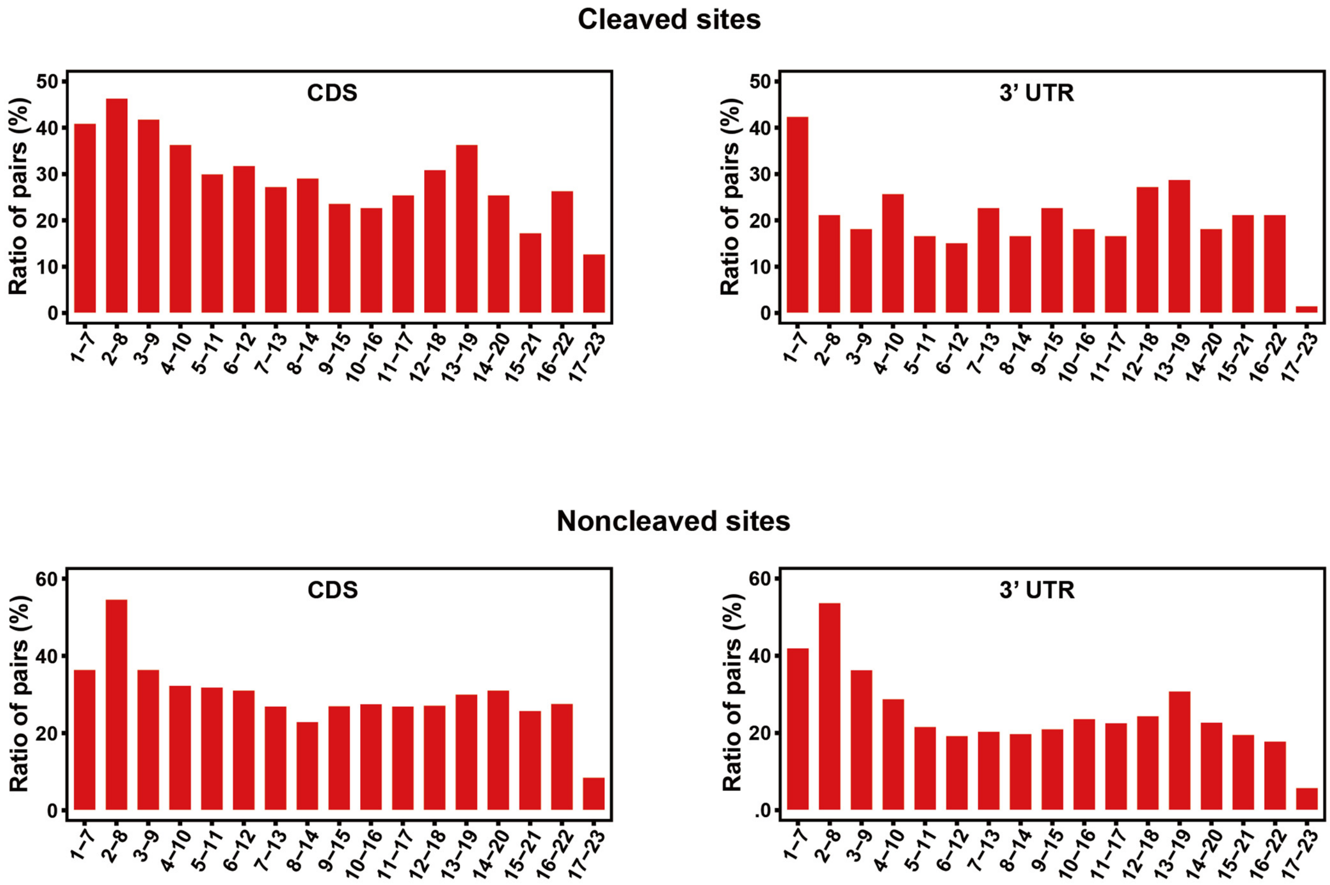

Figure S8. The miRNA-mediated cleaved and noncleaved sites exhibit diverse base-pairing rules

Base-pairing tendencies of different regions within miRNAs to their complementary sites within SpyCLIP identified miRNA-mediated cleaved (top panels) or noncleaved sites (bottom panels) located within the coding region or the 3' UTR. Each 7-mer region (nucleotides 1-7, 2-8, 3-9, and so on) of the AGO2-bound top 100 miRNAs was aligned with SpyCLIP-identified AGO2 cleaved or noncleaved target sites.

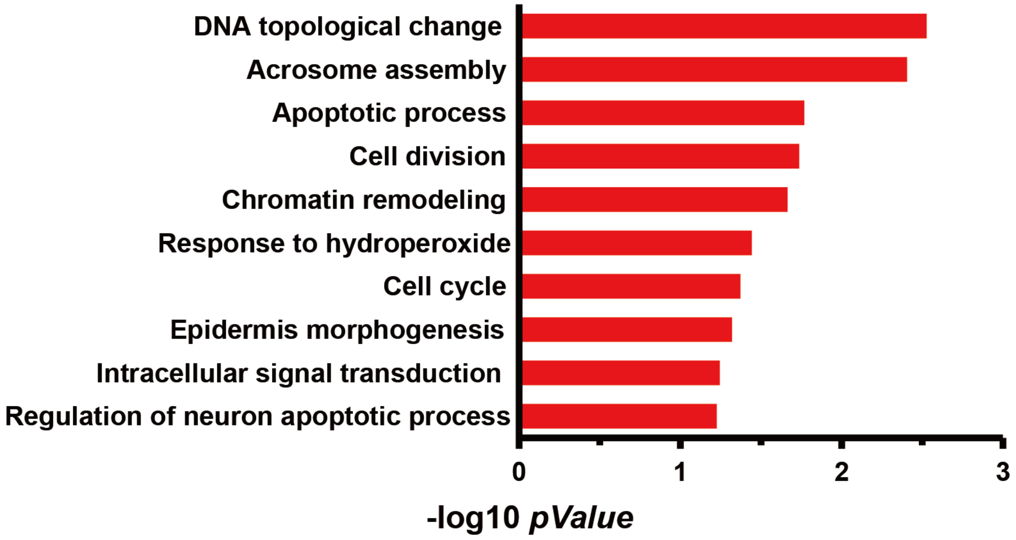

Figure S9. Gene ontology (GO) analysis of genes that are potentially cleaved by endogenous miRNAs

The potential AGO2-miRNA cleaved targets are enriched in the processes of DNA topological change, acrosome assembly, and several other critical cellular processes.
